# Medical Cannabis During Pregnancy and Breastfeeding: Is the Concern Fully Evidence-Based?

**DOI:** 10.3390/biomedicines14081703

**Published:** 2026-07-29

**Authors:** Miri Pevzner, Dror Sasson, Chen Porat, Daniel Porat, Arik Dahan

**Affiliations:** 1 Department of Clinical Pharmacology, School of Pharmacy, Faculty of Health Sciences, Ben-Gurion University of the Negev, Beer-Sheva 8410501, Israel; miripev@gmail.com (M.P.); drorsasson24@gmail.com (D.S.); porat86@gmail.com (D.P.); 2 Department of Obstetrics and Gynecology, Barzilai University Medical Center, Ashkelon 7830604, Israel; chene@bmc.gov.il

**Keywords:** medical cannabis, cannabinoids, Δ^9^-tetrahydrocannabinol (THC), cannabidiol (CBD), pharmacokinetics, maternal-fetal exposure, breast milk transfer, pregnancy and lactation, risk-benefit analysis

## Abstract

Medical cannabis use is steadily increasing worldwide, including among pregnant and breastfeeding women. This Perspective provides an overview of current evidence regarding cannabis exposure during pregnancy and lactation and its implications for clinical decision-making. The available literature indicates relatively consistent evidence linking prenatal Δ^9^-tetrahydrocannabinol (THC) exposure with adverse perinatal outcomes, including reduced birth weight, increased risk of preterm delivery, and potential neurodevelopmental effects, although the findings remain heterogeneous and often confounded. In contrast, evidence regarding cannabis exposure during breastfeeding is sparse, and long-term infant outcomes associated with postnatal exposure through breast milk remain insufficiently characterized. Data on isolated cannabidiol (CBD) exposure during pregnancy and lactation are particularly limited, and most available evidence derives from preclinical studies or pharmacokinetic modeling rather than clinical outcome studies. Current recommendations from major health organizations uniformly advise avoiding cannabis and cannabinoid use during pregnancy and breastfeeding. This Perspective highlights the need to interpret the evidence according to cannabinoid type, exposure period, route and pattern of use, and the distinction between recreational exposure and regulated medical use. Such distinctions are particularly important because evidence is relatively stronger for prenatal THC exposure, whereas data on CBD and lactational exposure remain limited and are derived largely from indirect clinical, pharmacokinetic, and preclinical sources. Within this evidence landscape, individualized clinical interpretation may be needed when exposure has already occurred, cessation is not immediately feasible, or maternal treatment decisions remain complex, while remaining anchored in current avoidance recommendations.

## 1. Introduction

Cannabis is an umbrella term for several plant species, including hemp and marijuana. Over the past two decades, its medical and recreational use has increased markedly worldwide, including among pregnant and breastfeeding women, who are the primary focus of this Perspective. This increase has occurred alongside growing social acceptance and expanding legalization of cannabis across many regions of the world [1,2,3,4,5,6,7]. Cannabinoids are a class of naturally occurring compounds found in cannabis that mediate the plant’s medicinal and psychoactive effects. They are absorbed through the lungs following inhalation or through the gastrointestinal tract after oral administration. The cannabinoids most commonly used for medical purposes are Δ^9^-tetrahydrocannabinol (THC) and cannabidiol (CBD) [8].

CBD has a wide range of potential medical applications and has become increasingly popular for the treatment of various conditions. Unlike THC, however, CBD does not produce euphoric or psychoactive effects [9]. CBD is a lipophilic compound derived from the *Cannabis sativa* plant, primarily from hemp varieties, which contain only trace amounts of THC (typically less than 0.3%). For medical use, CBD may be administered by inhalation (smoking or vaporization) or by oral formulations [10].

However, marijuana, which is typically characterized by a high THC content, raises particular concern because of its psychoactive effects, which may be harmful to both the mother and the neonate [11,12]. It is important to note that some pharmaceutical preparations of medical cannabis contain both THC and CBD. Therefore, accurate assessment of the specific product and its composition is essential for providing personalized clinical recommendations [13].

Although substantial information is available regarding the effects of cannabis use during pregnancy, data on its impact during breastfeeding remain limited [14]. This gap is clinically important because some women use cannabis or cannabinoid-containing products to manage symptoms such as anxiety, post-traumatic stress symptoms, chronic pain, sleep disturbance, nausea, or neurologic conditions such as multiple sclerosis [6,15,16]. In these complex clinical situations, the practical question is not whether cannabis should be recommended during lactation, but how clinicians should counsel patients when cannabis exposure is already occurring, when abrupt cessation is not feasible, or when maternal symptoms remain inadequately controlled despite evaluation of safer therapeutic alternatives.

Recent qualitative and mixed-methods studies indicate that clinical decision-making around cannabis use in pregnancy and lactation is complex and often inconsistent. A systematic review by Panday et al. reported substantial variability in clinicians’ knowledge and counseling practices, with many providers expressing uncertainty due to limited training and the absence of clear evidence-based guidelines [17]. Likewise, Vanstone et al. found that pregnant individuals frequently perceive cannabis as relatively safe and receive conflicting messages from healthcare professionals [18]. These findings highlight that beyond biological risk, gaps in knowledge and communication significantly influence how cannabis-related risks and benefits are perceived and discussed during pregnancy and breastfeeding.

The duration of breastfeeding and whether feeding is exclusive or partial are additional factors to consider when evaluating the risks associated with medical cannabis use during lactation. Current guidelines strongly recommend exclusive breastfeeding for the first six months postpartum. After this period, complementary foods are introduced and breast milk is no longer the infant’s sole source of nutrition [19]. In the clinical literature addressing drug safety during breastfeeding, infant age is recognized as an important determinant of exposure. Older infants are generally at lower risk because they breastfeed less frequently, exhibit more mature drug metabolism and clearance, and receive smaller volumes of breast milk as lactation gradually declines. Consequently, drug exposure through breast milk typically decreases as the infant grows [20]. Accordingly, these breastfeeding characteristics, together with the infant’s age, should be carefully considered when assessing drug exposure. Older infants who consume complementary foods and rely less on breast milk will inherently have reduced exposure to medications.

This Perspective provides a critical overview of the available evidence regarding cannabis use during pregnancy and lactation, with particular attention to the limited data concerning CBD exposure during breastfeeding. In addition, it discusses important clinical considerations and evidence gaps relevant to these populations. Finally, current guidelines and expert recommendations are reviewed to place the available evidence within a broader clinical context [21].

This article is intended as a clinically oriented Perspective rather than a systematic review or scoping review. Accordingly, it does not aim to provide a comprehensive or reproducible synthesis of all available studies on cannabis exposure during pregnancy and lactation. Instead, the literature was selected in a targeted, non-systematic manner to support a focused clinical discussion of key evidence, current recommendations, pharmacokinetic considerations, preclinical findings, and remaining evidence gaps. The specific contribution of this Perspective is to place the available evidence and current avoidance recommendations within a practical counseling context. This context distinguishes between THC-containing and CBD-dominant products, pregnancy and lactation, recreational exposure and regulated medical use, and population-level recommendations versus individualized counseling. Such counseling may be required when exposure has already occurred, when cessation is not immediately feasible, or when maternal treatment decisions require consideration of both infant safety and the potential consequences of untreated maternal disease or breastfeeding discontinuation.

## 2. THC Exposure During Pregnancy and Breastfeeding

The primary concern regarding cannabis use during pregnancy and lactation relates to THC exposure. THC is a small, highly lipophilic molecule that is rapidly distributed to the brain and adipose tissue. Its elimination half-life, following hepatic metabolism, ranges from approximately 20–36 h in occasional users to 4–5 days in heavy users, while complete excretion from the body may take up to 30 days [22,23]. THC acts as a partial agonist at cannabinoid type 1 (CB1) receptors and can bind to these receptors during fetal development, competing with endogenous cannabinoids. This interaction may disrupt normal endocannabinoid system signaling [24]. Notably, unlike in the adult brain, where CB1 receptors are widely distributed across most regions, in the fetal brain these receptors are predominantly localized to areas involved in emotional regulation, cognition, and memory [25,26].

### 2.1. THC Exposure During Pregnancy

Concerns regarding the potential effects of THC on the fetus and neonate initially arose from preclinical studies. In a rat model, THC was shown to cross the placenta and produce embryonic plasma concentrations reaching approximately 10% of maternal levels following a single exposure; with repeated exposure, embryonic plasma concentrations of THC were even higher [22,23].

Human fetal studies indicate that endocannabinoids, that is, cannabinoids that are produced endogenously in the body, play a crucial role in normal brain development. Cannabinoid receptors are present in the fetal central nervous system as early as the first 14 weeks of gestation, with receptor density increasing as pregnancy progresses. This pattern underscores the importance of endocannabinoid signaling in normal neurodevelopment [27,28,29,30]. It is also well established that cannabis use can affect the physiological function of the human placenta throughout pregnancy, including placental permeability and blood flow [24,31,32].

Investigating the effects of THC use on fetal development in humans is challenging because numerous confounding factors, such as low socioeconomic status, poverty, and malnutrition, can obscure effects specifically attributable to THC exposure. In addition, studies of cannabis use during pregnancy often rely on self-reported data regarding frequency, timing, and quantity of use, which may limit accuracy [33]. A 2025 Nature Communications study quantified Δ^9^-THC and its active metabolite 11-OH-THC concentrations in maternal plasma, placenta, fetal tissues, and umbilical venous plasma across all three trimesters. Using a validated maternal-fetal physiologically based pharmacokinetic (PBPK) model, the authors predicted fetal brain exposures under typical cannabis dosing. The model showed that although fetal exposure to Δ^9^-THC and 11-OH-THC is lower than maternal levels, fetal brain concentrations may peak early in gestation, providing quantitative insight into exposure dynamics that can better inform mechanistic studies of neurodevelopmental risk [34].

Cannabis use during pregnancy has been associated with a range of adverse outcomes, from reduced birth weight to impaired neurocognitive development in offspring, including deficits in executive function, verbal abilities, attention, and academic performance [35,36]. Reported neurodevelopmental and behavioral outcomes include impairments in attention, inhibitory control, executive function, visual-motor coordination, language or verbal abilities, social development, behavioral regulation, and school performance. Associations with broader neurodevelopmental or psychiatric outcomes, including developmental delay, psychotic-like experiences, and autism spectrum disorder (ASD), have also been investigated, although the findings are heterogeneous and often attenuated after adjustment for confounding factors [37,38,39,40]. Preclinical evidence supports these clinical observations.

Epigenetic mechanisms have been proposed as one possible explanation for the long-term neurodevelopmental effects associated with prenatal THC exposure. These mechanisms may include changes in DNA methylation, histone modifications, and non-coding RNA regulation, which can alter gene expression without changing the underlying DNA sequence [41,42,43]. Such changes are biologically plausible during fetal development, when neuronal differentiation, migration, synaptogenesis, and neurotransmitter-system maturation are tightly regulated. For example, prenatal cannabis exposure has been associated with reduced dopamine D2 receptor gene expression in the fetal amygdala, a brain region involved in emotional processing and reward-related behavior [25,44,45]. Because dopaminergic signaling contributes to reward processing, motivation, executive control, and susceptibility to substance use disorders, altered dopamine receptor expression has been proposed as one mechanism linking prenatal THC exposure with later behavioral and psychiatric vulnerability [46,47]. However, these mechanistic findings should be interpreted cautiously, as direct causal links between specific epigenetic changes and later clinical outcomes in humans remain incompletely established.

The effects of prenatal THC exposure on school performance are less well defined. Findings across studies are inconsistent, largely because it is difficult to disentangle the direct impact of THC exposure from the influence of the postnatal environment and other confounding factors related to the child’s upbringing [48,49,50]. Nevertheless, several studies have reported that children exposed to THC in utero exhibit lower performance on tests of visual problem-solving, visual-motor coordination, and visual analysis, as well as reduced attention span and increased behavioral difficulties compared with unexposed children [51,52].

In a contemporary prospective cohort study, prenatal cannabis exposure was associated with poorer performance on laboratory-based measures of executive function (such as attention, inhibitory control, and planning) and with increased observed aggressive behavior at age 5 years in exposed children compared with unexposed peers. These findings were robust to adjustment for confounders and highlight specific cognitive and behavioral domains affected early in childhood that are relevant to academic success and adaptive functioning [53]. Another recent investigation reported that cannabis exposure occurring after maternal recognition of pregnancy was associated with a small but measurable increase in the risk of psychopathology during childhood; this relationship persisted after controlling for multiple potential confounders, including in analyses of psychotic-like experiences [54].

Prenatal THC exposure has also been linked to impaired fetal growth. A retrospective cohort study reported an increased risk of birth weight below the 10th percentile among cannabis users [55]. In addition, multiple studies have documented smaller head circumference and lower birth weight in exposed newborns [23,56,57,58], with these associations appearing more pronounced among women who are heavy users of cannabis, particularly during the first and second trimesters of pregnancy [58,59,60]. More recently, large cohort studies have confirmed these risks. For example, one analysis found that newborns prenatally exposed to cannabis weighed on average 218 g less than non-exposed infants, and had higher rates of low birth weight, preterm delivery, neonatal intensive care unit (NICU) admission, and lower Apgar scores [61]. Another population-based study reported that in utero cannabis exposure was associated with an increased likelihood of adverse neonatal outcomes, including low birth weight, small-for-gestational-age status, preterm delivery, and NICU admission, and demonstrated a dose-response relationship, with more frequent prenatal cannabis use linked to greater risk of growth restriction [62]. By contrast, a longitudinal analysis from the ALSPAC cohort found only modest associations between maternal cannabis use and perinatal outcomes (and later educational achievement), and these were substantially attenuated after controlling for socioeconomic and familial confounding; notably, similar patterns were observed for paternal cannabis use, suggesting that residual confounding rather than direct biological effects might explain some of the associations reported in other studies [63].

Evidence regarding early childhood developmental milestones after prenatal cannabis exposure has been mixed. Gutiérrez Álvarez et al. conducted a retrospective analysis of routine well-child visits and found that prenatal THC exposure was associated with an increased risk of developmental delays in infancy, particularly in the fine motor and social domains [64]. In contrast, a large population-based cohort of nearly 120,000 mother-child pairs found that early prenatal cannabis use (primarily in the first trimester) was not associated with an increased risk of early developmental delays, such as speech or language disorders, motor delays, or global developmental delays, through age 5.5 years. Although this study suggests null findings for certain early childhood outcomes, the authors emphasized the need for research on cannabis use later in pregnancy, patterns of exposure, and longer-term outcomes [65].

Polysubstance use is another important consideration. In pregnancy, cannabis exposure frequently co-occurs with other substances, particularly tobacco or nicotine products, and in some populations also with alcohol, opioids, or other psychoactive substances. This co-use is clinically important for two reasons. First, combined exposures may increase fetal and neonatal risk beyond the effect of cannabis alone. Second, polysubstance use makes causal interpretation more difficult, because adverse outcomes attributed to cannabis may partly reflect the independent or additive effects of nicotine, alcohol, or other exposures. A recent large population-based cohort study found that prenatal co-exposure to cannabis and nicotine products was associated with a higher risk of adverse perinatal outcomes, including small-for-gestational-age infants, preterm delivery, neonatal morbidity, and neonatal death, compared with exposure to either substance alone [66]. These findings highlight the need for counseling that addresses cannabis together with tobacco/nicotine and other substance exposures, rather than discussing cannabis in isolation. Additionally, a retrospective analysis of more than 316,000 pregnancies reported that maternal cannabis use was associated not only with adverse neonatal outcomes but also with elevated maternal risks, including gestational hypertension, preeclampsia, placental abruption, and abnormal gestational weight gain. These findings indicate that cannabis exposure during pregnancy may affect both fetal and maternal health, further supporting cautious clinical counseling [67].

In summary, the current recommendation to avoid all THC use during pregnancy is strongly supported by robust evidence. Notably, Solmi et al. evaluated the credibility and certainty of evidence regarding cannabis, cannabinoids, and cannabis-based medicines by analyzing 101 meta-analyses of randomized controlled trials and observational studies, and their umbrella review likewise supported the avoidance of cannabis use during pregnancy [68].

### 2.2. THC Exposure During Breastfeeding

Beyond concerns related to pregnancy, the potential effects of THC exposure on the breastfed infant also warrant careful consideration. When cannabis is used during both pregnancy and breastfeeding, it may be difficult to distinguish whether any observed effects in the infant are attributable to prenatal exposure or to postnatal exposure through breast milk (or both). In addition, in the context of maternal cannabis use, the potential harm associated with infant exposure to smoke/secondhand aerosols should be taken into account, and not solely exposure via breast milk [69,70].

The secretion of drugs into human milk depends on their physicochemical properties, including ionization state, molecular weight, lipid solubility, and pH [71]. THC is highly lipophilic and has a low molecular weight; therefore, it readily passes into breast milk [72,73]. Owing to these properties, THC accumulates in adipose tissue and is released gradually into the systemic circulation and breast milk over a prolonged period [74]. THC may be detectable in human milk as early as 1–4 h after a single use and for up to 6 days following the last reported use [74,75,76], and in some cases even up to 3–6 weeks later with heavy chronic use [77]. Consequently, the practice of “pumping and dumping” (expressing and discarding breast milk after cannabis use in an effort to reduce infant exposure) is unlikely to be effective. The mean maximal concentration of THC in breast milk has been reported to occur approximately 1 h after maternal inhalation of cannabis, followed by a gradual decline each hour thereafter [74]. One commonly used method for estimating drug exposure during breastfeeding is the relative infant dose (RID), defined as the dose received by the infant through breast milk (mg/kg/day) divided by the maternal dose (mg/kg/day) [78].

Baker et al. demonstrated that the concentration of THC in breast milk depends on the maternal daily dose. Among occasional users, approximately 2.5% of the maternal THC dose is transferred to breast milk. In contrast, in heavy users, THC concentrations in milk may be substantially higher, on the order of 8% or more, due to accumulation of THC in maternal tissues. Thus, maternal frequency of use is a critical determinant of infant THC exposure [79]. A study published in 2018 evaluated the extent of THC transfer into breast milk in women who used cannabis occasionally versus frequently. That study also suggested a potential effect of cannabis on lactation itself, demonstrating that its exposure may reduce breast milk production, possibly through suppression of prolactin secretion [72].

Although direct empirical evidence in pregnant or lactating humans is limited, the findings from animal studies and from studies in nonpregnant, nonlactating individuals suggest that long-term accumulation of cannabinoids in mammary adipose tissue may interfere with lactogenesis through direct activation of cannabinoid receptors [80,81]. In addition, phytocannabinoids may influence milk output and composition, particularly lipid and fatty acid content, by modulating the expression of genes involved in fatty acid synthesis. Cannabinoids may also affect the regulation of milk synthesis and secretion through interactions with the endocrine system. Nevertheless, substantial research using rigorous methods for milk collection and measurement of milk production is required to confirm these potential effects [80]. Furthermore, one report noted that maternal cannabis use might reduce secretory immunoglobulin A (sIgA) levels in human milk, hinting at potential immunologic impacts [82]. Overall, far less information exists about cannabis use during breastfeeding than during pregnancy. Some reports indicate that infants exposed to THC via breast milk may experience short-term effects such as sedation, lethargy, and poor feeding behaviors [83,84,85].

One older study in 1990 suggested that THC exposure through breast milk in the first month of life was associated with delayed motor development at one year of age, in a dose-dependent manner [86]. However, this study had a very small sample size. Additionally, it evaluated cannabis exposure during the 1980s, and given the much higher potency of modern cannabis [87], contemporary studies are needed to determine whether developmental outcomes differ following exposure to today’s more potent cannabis products [88]. To date, no well-designed studies have examined the long-term neurodevelopmental outcomes of infants exposed to THC via breast milk. Table 1 summarizes selected recent and clinically relevant original human studies illustrating key findings on THC exposure during pregnancy and lactation. The table is not intended to represent a systematic or exhaustive summary of all the available studies.

## 3. Cannabidiol (CBD) Influence

**Scheme 1:**
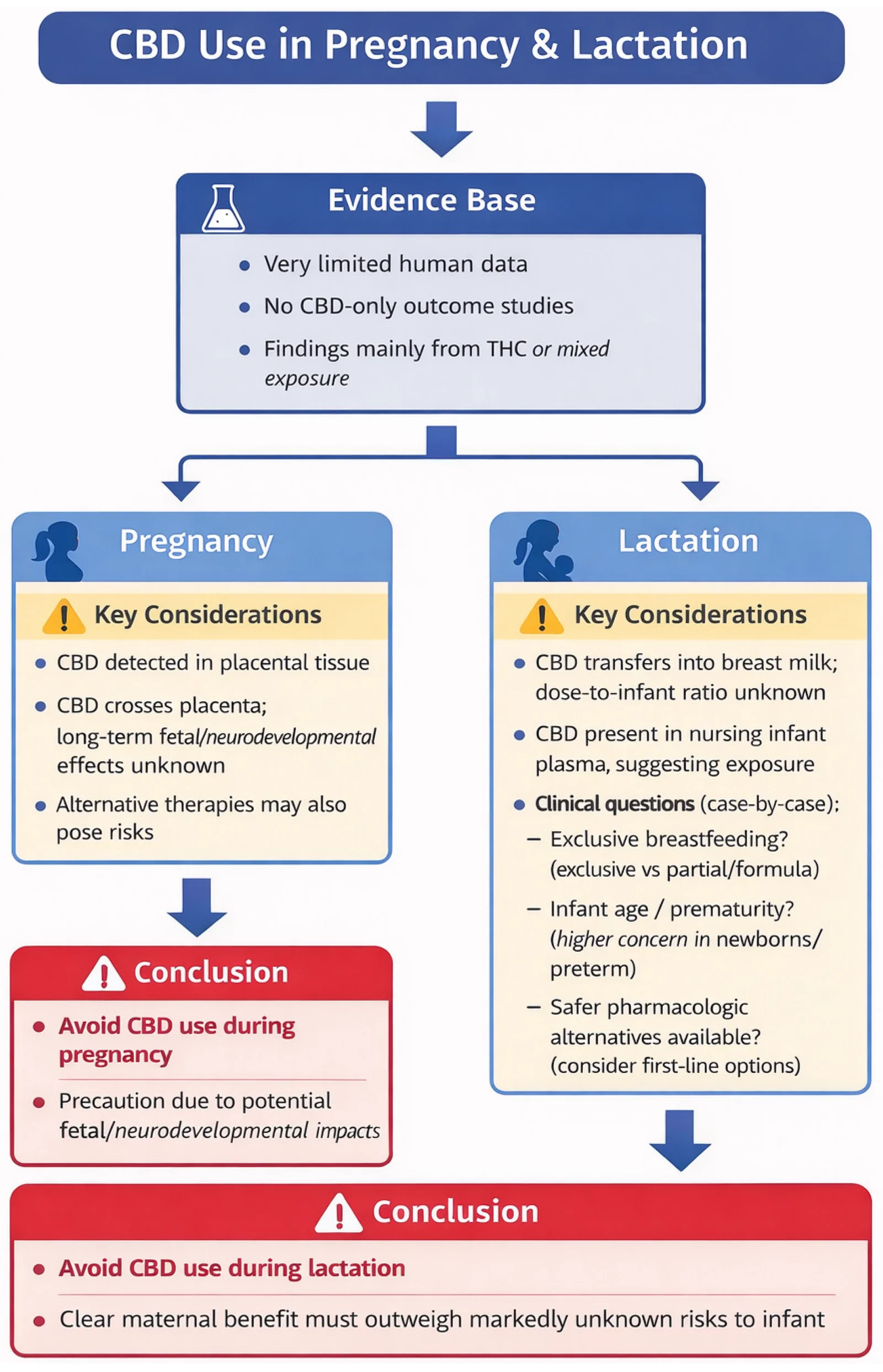
**Key considerations related to CBD exposure during pregnancy and breastfeeding.** The scheme summarizes the main clinical questions regarding CBD exposure in pregnancy and lactation, including placental or breast milk transfer, limited human outcome data, infant age, breastfeeding intensity, and the maternal treatment context. CBD, cannabidiol.

Assessing the risks associated with CBD use, on the one hand, and the implications of avoiding its use, on the other, is considerably more complex when (albeit infrequent) medical cannabis preparations contain CBD exclusively. CBD has potential anti-inflammatory, analgesic, and antiemetic properties, and its lack of psychoactive effects, together with a perception of relative safety, has led many pregnant women to use this compound [15]. While the effects of THC on pregnancy have been extensively investigated, the literature addressing CBD use during pregnancy remains limited. To date, most studies have not isolated or specifically examined the effects of CBD on the developing fetus, the pregnant mother, or the breastfed infant [89]. The distinct considerations involved in CBD use during pregnancy and breastfeeding are illustrated in Scheme 1.

### 3.1. CBD Pharmacokinetics

With growing public interest in CBD, various formulations have been developed for multiple routes of administration, including oral ingestion, oromucosal delivery, inhalation, transdermal application, and even intravenous administration [90]. An important characteristic of CBD is its high lipophilicity, which accounts for its frequent formulation in oil-based preparations. Some evidence suggests that taking CBD in the fed state or together with fatty foods may enhance its absorption. In addition, cannabinoids readily accumulate in adipose tissue and dissipate slowly over time [89,91]. Pharmacokinetic studies have demonstrated that the elimination half-life of CBD after a single dose is approximately 1 day following intravenous administration, 31 h after inhalation, and 2–5 days after chronic oral ingestion in humans [92]. The bioavailability of CBD varies considerably depending on the route of administration and between individuals. Across studies, oral bioavailability ranged from about 13% to 19%, whereas inhalational bioavailability ranged from 11% to 45%. In pregnant mice, CBD has shown much shorter half-lives: following a single dose, maternal and fetal elimination half-lives were approximately 5 and 2 h, respectively, with moment analysis indicating a mean residence time of less than 2 h in both the mother and fetus [89,93].

### 3.2. CBD Influence During Pregnancy

In vivo studies suggest that CBD may alleviate certain adverse effects of prenatal THC exposure and other prenatal insults. In a Wistar rat model, offspring exposed to THC in utero showed deficits in hippocampal synaptic plasticity and endocannabinoid signaling, which were associated with cognitive impairment; administration of CBD to the adolescent offspring improved their cognitive performance [94]. In another study using the same rat strain, offspring of mothers fed a high-fat/high-sugar diet during pregnancy developed metabolic and inflammatory disturbances; oral CBD treatment in these offspring reduced pro-inflammatory markers (tumor necrosis factor-α, interleukin-6, and interleukin-1β), decreased white adipose tissue mass and triglyceride levels, and reversed insulin resistance [95]. Likewise, in a mouse model of maternal immune activation (a proxy for prenatal viral infection), CBD administration effectively mitigated behavioral abnormalities in the offspring by reducing glutamatergic transmission and enhancing GABAergic neurotransmission in pyramidal neurons of the medial prefrontal cortex [96]. CBD has also shown benefits in a model of fetal alcohol spectrum disorder: in a recent study, female mice were exposed to ethanol from gestational day 7 through postnatal day 21, and subsequent prolonged CBD treatment (for 4–6 weeks) was found to partially normalize the ethanol-induced emotional and cognitive disturbances, as well as associated gene expression and cellular changes, with some differences observed between male and female offspring [97].

Human data on isolated CBD exposure during pregnancy are extremely limited. In one longitudinal study, a small subset of infants (3 of 15) had concurrent prenatal CBD exposure detectable in mid-gestation, suggesting that CBD can cross the placenta; however, the sample size was too small to draw definitive conclusions about CBD’s independent effects on growth, underscoring the need for larger datasets to clarify CBD-specific developmental outcomes [98]. Ex vivo human placental perfusion studies have confirmed that the placenta can significantly limit fetal exposure to CBD. Berman et al. showed that the placenta functions as a depot compartment for CBD, with concentrations in the fetal (umbilical) circulation approximately one-fifth of those in the maternal circulation [99]. In addition, in vitro studies examining the effects of CBD on placental cells have shown that CBD can interfere with trophoblast cell turnover and placental remodeling [100,101].

Animal studies have also examined how CBD distributes to and affects the developing brain. In one study, fetal, postnatal, and adult rats were administered CBD, and it was found that CBD rapidly penetrated both the developing and adult brain, whereas entry into the cerebrospinal fluid was more limited. Consistent with the findings of Berman et al., placental transfer of CBD was substantially restricted, with fetal plasma concentrations reaching roughly 50% of maternal plasma levels. Albumin was identified as the primary (though not exclusive) binding protein for CBD at all developmental stages [102].

Emerging evidence suggests prenatal CBD exposure can have subtle, sex-dependent effects on neurodevelopment. In one mouse study, pregnant dams were treated with CBD from embryonic day 5 until delivery. Prenatal CBD exposure was found to increase sensitivity to thermal pain in adult male offspring via TRPV1 (transient receptor potential vanilloid 1) signaling. In addition, female offspring exposed to CBD in utero showed impaired problem-solving behavior and electrophysiological alterations in layer 2/3 pyramidal neurons of the prefrontal cortex, including an increased current threshold to elicit action potentials and a reduced number of action potentials [103]. The insular cortex is a brain region involved in processing hunger, pain, fatigue, and emotional states such as disgust, fear, anxiety, and happiness [104]. Prenatal exposure to CBD has been shown to disrupt neuronal development in the insular cortex, leading to a loss of the normal functional distinctions between its major subdivisions [105].

### 3.3. CBD Influence During Breastfeeding

Cannabidiol has not been systematically studied in nursing women using pharmaceutical CBD preparations. Nevertheless, it has been detected in the breast milk of some mothers who used cannabis products. One report found that CBD is excreted into human breast milk in very small amounts [106]. Similarly, another analysis detected CBD in the milk of women who reported using cannabis during lactation [80]. Consistent with these findings, a prospective study measuring cannabinoids in plasma and breast milk of breastfeeding mothers confirmed that both THC and CBD accumulate in breast milk relative to maternal plasma, and it noted that cannabis use frequency often increases during the early postpartum period [107]. In addition, in vitro studies suggest that cannabinoids, including CBD, may alter the macronutrient composition of human milk (for example, affecting levels of certain milk proteins and lipids) [108,109]. Furthermore, a 2023 physiologically based pharmacokinetic modeling study using real-world breast milk cannabinoid concentrations found that even at the high end of simulated exposure scenarios, the estimated systemic CBD dose in breastfed infants is substantially lower than doses used therapeutically in pediatric patients. However, the authors emphasized that variability related to maternal use patterns and administration routes remains a key uncertainty [110], underscoring the need for randomized pharmacokinetic studies to better characterize exposure. Table 2 summarizes selected original human and translational studies relevant to CBD exposure during pregnancy and lactation. The table is intended to illustrate the limited and heterogeneous evidence base rather than provide a systematic or exhaustive summary.

### 3.4. CBD Influence on Pediatrics

Beyond pregnancy and lactation, it is noteworthy that CBD has demonstrated therapeutic benefits in certain pediatric populations. Several clinical trials and reviews have reported significant reductions in seizure frequency with medical CBD treatment in children with refractory epilepsy syndromes such as Dravet syndrome, with some patients achieving complete seizure freedom. On average, treatment with CBD has been associated with approximately a 50% reduction in seizure frequency and an improved caregiver Global Impression of Change compared with placebo, and the overall safety profile of CBD in these trials was considered acceptable (most adverse events were manageable) [111,112]. The U.S. Food and Drug Administration (FDA) has approved Epidiolex^®^, a pharmaceutical formulation containing purified CBD, for the treatment of seizures associated with Lennox-Gastaut syndrome and Dravet syndrome in patients aged 1 year and older, as well as for seizures associated with tuberous sclerosis complex in patients 1 year of age or older [113,114]. These approvals indicate that the FDA has concluded this specific product to be safe and effective for its approved indications. In addition, recent studies in children with ASD have reported improvements in hyperactivity, anxiety, sleep disturbances, and self-injurious behaviors following CBD treatment [115]. However, CBD is not without adverse effects. Commonly reported side effects in pediatric use include somnolence, fatigue, and diarrhea, and there are case reports suggesting that CBD might paradoxically precipitate seizures in some toddlers [116,117].

## 4. Preclinical Evidence

Preclinical evidence is important for interpreting the human literature because it provides biological plausibility for associations that are difficult to evaluate causally in observational studies. Animal and cellular models allow controlled assessment of cannabinoid exposure during defined developmental windows and can help clarify potential mechanisms involving placental function, endocannabinoid signaling, neurodevelopment, lactation biology, and cardiovascular development. However, these findings should be interpreted as mechanistic support rather than direct evidence of clinical risk in humans [118]. Consistent with this mechanistic role, a growing body of preclinical evidence supports potential adverse effects of prenatal exposure to THC on fetal neurodevelopment (and even cardiovascular development). Experimental studies have demonstrated that THC exposure during gestation can disrupt neuronal migration, synaptogenesis, and cortical circuit formation, and is associated with long-lasting alterations in neurotransmitter systems, stress responsivity, and cognitive performance in offspring [24,119,120,121]. By contrast, there is limited direct experimental evidence on the effects of isolated prenatal CBD exposure on fetal brain development. CBD is known to cross the placenta and reach the fetus, but few studies have specifically evaluated its long-term impact on brain development. This lack of evidence highlights a critical knowledge gap and underscores the need for dedicated research into prenatal CBD safety.

Recent preclinical studies also suggest that prenatal cannabinoid exposure may have implications for cardiovascular development. In a rat model, maternal exposure to THC during pregnancy was associated with fetal growth restriction and impaired cardiac function in the offspring, including reduced heart size at birth, postnatal catch-up growth, and molecular markers of adverse cardiac remodeling during early life, findings that indicate potential long-term cardiometabolic vulnerability [122]. More recently, emerging data have begun to address the potential cardiac effects of prenatal CBD exposure. A 2023 animal study reported that gestational CBD exposure was associated with reduced cardiac function in male offspring at three weeks of age, along with alterations in myocardial endocannabinoid signaling, despite preserved heart-to-body weight ratios [123]. Although these findings are preliminary and derived from animal models, they underscore how little is known about the developmental effects of CBD.

## 5. Current Expert Recommendations

Owing to the limited research available, current data are insufficient to adequately evaluate the effects of medical cannabis use on breastfed infants [41,124]. Consequently, most health organizations in the United States recommend avoiding the use of medical cannabis, including both THC and CBD, during pregnancy and breastfeeding [16,125].

The 2022 policy statement of the American Academy of Pediatrics (AAP), “Breastfeeding and the Use of Human Milk,” concluded that current data are insufficient to assess the effects of infant exposure to maternal cannabis use during breastfeeding and, therefore, discouraged medical cannabis use during lactation [20]. Similarly, the LactMed database (which summarizes professional guidelines) recommends that mothers be encouraged to avoid cannabis use while breastfeeding in order to reduce infant exposure, including exposure to cannabis smoke [106]. The American College of Obstetricians and Gynecologists (ACOG) has recently updated its guidance on cannabis use during pregnancy and lactation. ACOG continues to recommend that pregnant individuals, those contemplating pregnancy, and lactating women avoid cannabis use and be counseled regarding potential risks and safer alternatives. Importantly, the updated guidance also recognizes that continued cannabis use is not, by itself, a contraindication to breastfeeding. This distinction is clinically relevant because it supports avoidance as the default recommendation while still allowing individualized counseling and lactation support when cannabis use is disclosed or ongoing [16,106,126]. The U.S. Centers for Disease Control and Prevention (CDC) notes that the potential health effects of CBD use during pregnancy are currently unknown; nevertheless, the CDC advises avoiding cannabis use during both pregnancy and breastfeeding [127]. In addition, the FDA currently recommends avoiding the use of CBD during pregnancy and lactation because of insufficient safety data [128].

**Scheme 2:**
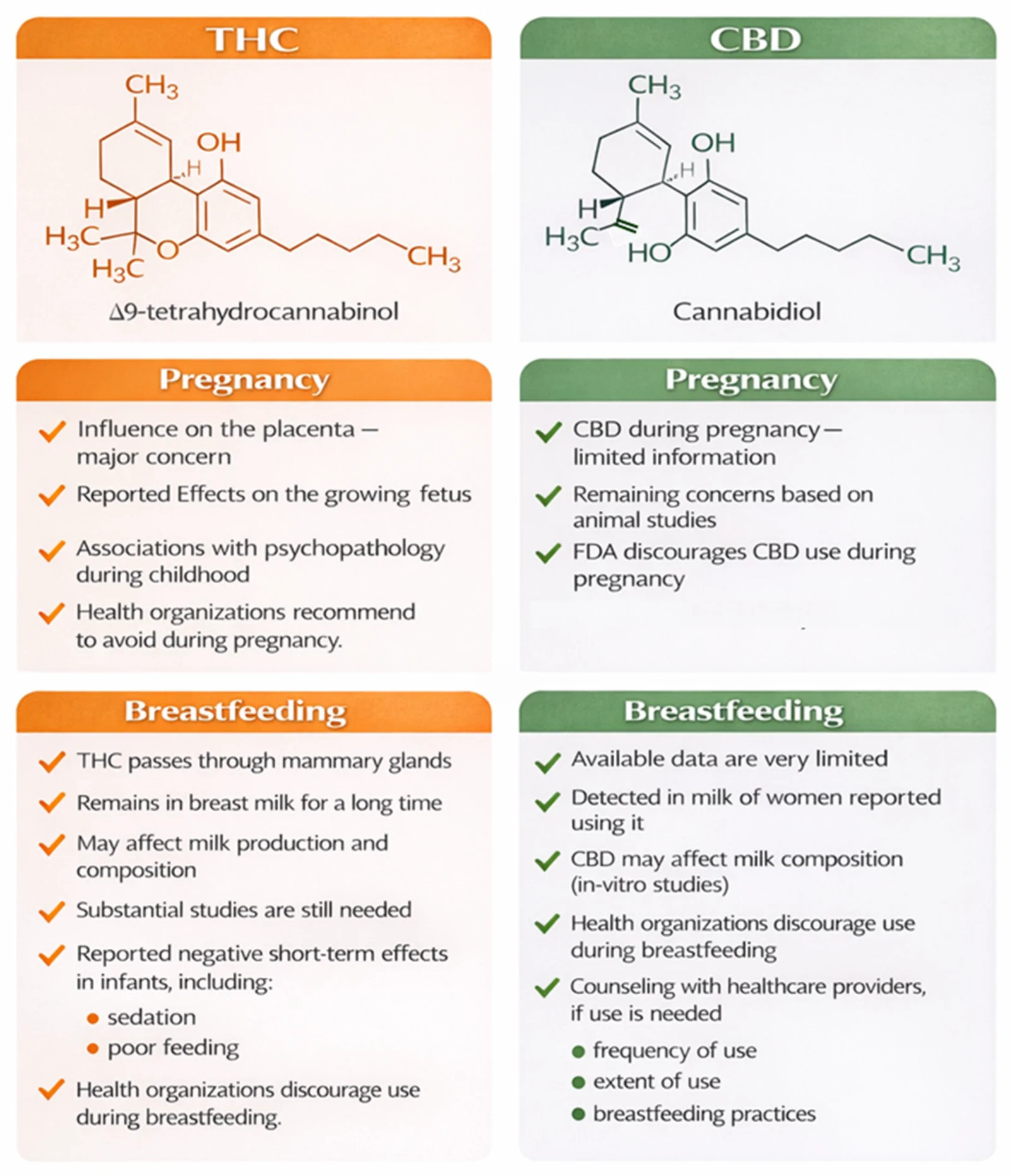
**Comparison of THC- and CBD-related considerations during pregnancy and breastfeeding.** The scheme contrasts THC- and CBD-related concerns according to exposure period, available evidence, transfer to the fetus and breastfed infant, and current avoidance recommendations. THC, Δ^9^-tetrahydrocannabinol; CBD, cannabidiol.

Finally, the Academy of Breastfeeding Medicine (ABM) emphasizes the need for further research into the long-term effects of cannabis use during breastfeeding, given that the currently available evidence remains limited. The ABM also recommends that healthcare providers offer appropriate counseling and guidance regarding cannabis use during lactation [126]. A comparison of THC and CBD use during pregnancy and breastfeeding is illustrated in Scheme 2.

Overall, current recommendations support avoidance of cannabis and cannabinoid use during pregnancy and breastfeeding as the default position. At the same time, the limited evidence base during lactation means that clinicians may still need to provide individualized interpretation when cannabis use is disclosed, exposure has already occurred, or maternal treatment decisions are clinically complex.

## 6. Discussion

Further research on medical cannabis use during pregnancy and breastfeeding is critically needed, particularly with respect to CBD, including randomized pharmacokinetic studies, dose-response evaluations, and infant outcome tracking. Current recommendations regarding CBD reflect the limited availability of human data, as well as evidence derived from animal studies and biological plausibility considerations. The increasing prevalence of medical cannabis use highlights the need for evidence-based guidelines that explicitly distinguish between THC and CBD. Importantly, for certain maternal conditions, treatment decisions during pregnancy and lactation often involve weighing potential risks and benefits among imperfect therapeutic options, as several available treatments may also carry safety considerations for the fetus or breastfed infant [129].

It is also important to recognize that breastfeeding is considered the normative standard for infant feeding and nutrition, due to its well-established short- and long-term benefits for both mother and infant [130]. The AAP and the World Health Organization (WHO) recommend exclusive breastfeeding for approximately six months, with the introduction of complementary foods around six months of age, and continued breastfeeding thereafter (up to two years of age or longer) as mutually desired by the mother and child. Therefore, when considering cannabis exposure through human milk, potential risks to the infant should be evaluated alongside the recognized benefits of breastfeeding. This issue may become particularly relevant when maternal treatment is considered necessary, as decisions regarding medication use can sometimes affect breastfeeding continuation [131,132]. In addition, conception should ideally be postponed until cannabis use is discontinued, given potential developmental risks during early gestation.

To make individualized counseling more actionable, key clinical factors that may guide risk communication during lactation are summarized in Box 1.

Box 1 **Clinical decision-support considerations for cannabis exposure during lactation.**

  When counseling is required in the setting of ongoing or recent cannabis exposure during breastfeeding, several practical factors should be assessed:

  **Product composition:** THC-containing, CBD-dominant, or mixed cannabinoid product.

  **Pattern of use:** single or occasional exposure versus chronic, frequent, or heavy use.

  **Route of administration:** inhaled, vaporized, oral, or other formulations.

  **Exposure beyond milk:** potential infant exposure to secondhand smoke or aerosols.

  **Breastfeeding intensity:** exclusive breastfeeding versus partial breastfeeding or breastfeeding of an older infant receiving complementary foods.

  **Infant vulnerability:** newborn age, prematurity, low birth weight, medical fragility, or feeding difficulties.

  **Maternal indication:** severity of the maternal condition, risk of untreated disease, and previous response to safer therapeutic alternatives.

  **Feasibility of cessation or reduction:** including the need for psychiatric, addiction-medicine, lactation, or clinical pharmacology support.

  **Specialist consultation:** teratology information service, lactation specialist, obstetrician, pediatrician, or clinical pharmacologist, when available.

Interpretation of the human observational literature requires particular caution. Associations between prenatal cannabis exposure and perinatal or neurodevelopmental outcomes may reflect, at least in part, confounding by socioeconomic factors, maternal mental health, nutrition, tobacco or nicotine use, alcohol use, and other substance exposures. Familial and environmental factors may also contribute, as suggested by studies in which associations were attenuated after adjustment for socioeconomic or familial characteristics, or where similar patterns were observed for paternal cannabis use. At the same time, residual confounding does not exclude a biological contribution of cannabis exposure, particularly when associations are supported by dose-response patterns, timing of exposure, pharmacokinetic evidence of fetal exposure, placental effects, and preclinical data showing disruption of endocannabinoid signaling during development. Therefore, the available evidence should be interpreted neither as definitive proof of causality for all the reported outcomes nor as reassurance of safety.

The primary concern regarding medical cannabis use during lactation stems from evidence demonstrating adverse effects of THC exposure during pregnancy and early childhood. Additional concerns arise from the limited studies involving lactating women; however, when cannabis is used during pregnancy, it is often challenging to disentangle whether observed infant effects are due to prenatal exposure versus exposure via breast milk.

There is substantially less evidence-based data on the effects of cannabis exposure in breastfed infants than on prenatal exposure, particularly with respect to CBD, which has been scarcely studied in either pregnancy or lactation. Notably, in the most recent edition of Hale’s Medications & Mothers’ Milk, a distinction is made between chronic and infrequent maternal cannabis use. Chronic use is classified as “limited data-possibly hazardous,” whereas infrequent use is categorized as “limited data-probably compatible” [72]. This distinction may be useful for risk stratification when exposure has already occurred, but it should not be interpreted as a recommendation to initiate or continue cannabis use during breastfeeding. Rather, it underscores the importance of considering exposure pattern, product composition, and infant vulnerability when counseling lactating women.

## 7. Limitations

As this article is a Perspective and not a systematic or scoping review, the literature selection was targeted rather than exhaustive. Consequently, the studies discussed and summarized in the tables should be interpreted as selected examples of clinically relevant evidence rather than as a complete synthesis of all the available publications. This approach may introduce selection bias, but it supports the article’s aim: a focused discussion of key clinical distinctions, current recommendations, and evidence gaps. This Perspective is limited by the heterogeneity of the underlying literature and by the predominance of observational studies that are vulnerable to confounding (e.g., socioeconomic factors and co-use of nicotine, alcohol, or other substances) and exposure misclassification (self-report, inconsistent product characterization, and variable potency). Many studies do not distinguish THC from CBD or quantify dose, timing, and route of administration, which constrains causal interpretation and limits translation to counseling. For CBD, the paucity of human outcome data, particularly during breastfeeding, means that current guidance relies heavily on indirect evidence and precaution. It should be noted that most available human data derive from studies of recreational cannabis exposure rather than regulated medical cannabis use. Accordingly, extrapolation of these findings to clinical contexts of prescribed medical cannabis should be interpreted with caution.

## 8. Conclusions

Evidence supporting the avoidance of THC-containing products during pregnancy is relatively strong, although interpretation of specific outcomes remains limited by confounding, exposure misclassification, and polysubstance use. Evidence regarding CBD exposure, particularly during lactation, is far more limited, and current recommendations therefore continue to advise against cannabis and cannabinoid use during pregnancy and breastfeeding. At the same time, clinicians may encounter situations in which exposure has already occurred, cessation is not immediately feasible, or maternal treatment decisions are complex. In such cases, counseling should remain anchored in the recommendation to avoid use, while incorporating product composition, pattern and route of use, infant age and vulnerability, breastfeeding intensity, maternal condition, and the availability of safer therapeutic alternatives. Consultation with a teratology information service, lactation specialist, pediatrician, or clinical pharmacologist may be valuable when available.

## Figures and Tables

**Table 1:** Selected recent and clinically relevant original human research on THC exposure during pregnancy and lactation. THC, Δ^9^-tetrahydrocannabinol; ASD, autism spectrum disorder; LBW, low birth weight; SGA, small for gestational age; PTB, preterm birth.

#	Title	Year	Journal	Study Design	Cannabinoid	Main Findings	Interpretation	Ref.
1	Prenatal Cannabis Use and Maternal Pregnancy Outcomes	2024	JAMA Internal Medicine	Retrospective cohort	THC	Adverse maternal outcomes	Suggests clinically relevant risk	[67]
2	Risk of Adverse Neonatal Outcomes After Combined Prenatal Cannabis and Nicotine Exposure	2024	JAMA Network Open	Retrospective cohort	THC	Higher neonatal risk	Supports additive harm of cannabis and nicotine	[66]
3	Prenatal Cannabis Exposure and Executive Function and Aggressive Behavior at Age 5 Years	2024	JAMA Pediatrics	Prospective cohort	THC	Executive dysfunction	developmental concern	[53]
4	Early Maternal Prenatal Cannabis Use and Child Developmental Delays	2024	JAMA Network Open	Population cohort	THC	No association with child developmental delays	Does not confirm harm	[65]
5	Maternal Prenatal Cannabis Use and Child Autism Spectrum Disorder	2024	JAMA Network Open	Retrospective cohort	THC	No ASD association	No evidence for ASD risk	[40]
6	Neonatal outcomes associated with in utero cannabis exposure: a population-based retrospective cohort study	2024	American Journal of Obstetrics & Gynecology	Retrospective cohort	THC	Higher likelihood for LBW, SGA, PTB	Consistent adverse perinatal signal	[62]
7	Maternal cannabis use in pregnancy, perinatal outcomes, and cognitive development in offspring	2025	European Journal of Epidemiology	Nationwide cohort	THC	Reduced birth size	Dose-response supports causality	[63]
8	Persistence of Δ^9^-tetrahydrocannabinol in Human Breast Milk	2021	JAMA Pediatrics	Prospective, observational PK study	THC	Prolonged milk persistence	Infant exposure likely even after cessation	[77]
9	Human Milk Cannabinoid Concentrations and Associations with Maternal Factors: The Lactation and Cannabis (LAC) Study	2024	Breastfeeding Medicine	Prospective, observational PK study	THC	Variable milk exposure	High variability; individual risk assessment needed	[76]
10	Quantification and prediction of human fetal Δ^9^-tetrahydrocannabinol/(±)-11-OH- Δ^9^-tetrahydrocannabinol exposure during pregnancy to inform fetal cannabis toxicity	2025	Nature Communications	PBPK modeling	THC	Quantitative fetal exposure	Confirms biologically plausible fetal exposure	[34]

**Table 2:** Selected original human and translational studies relevant to CBD exposure during pregnancy and lactation. PK, pharmacokinetic; CBD, cannabidiol; THC, Δ^9^-tetrahydrocannabinol.

#	Title	Year	Journal	Study Design	Cannabinoid(s)	Main Contribution	Interpretation	Ref.
1	Cannabis use and measurement of cannabinoids in plasma and breast milk of breastfeeding mothers	2021	Pediatric Research	PK study	CBD + THC	CBD transfer into milk	Exposure confirmed; outcomes unknown	[107]
2	Cannabidiol Exposure Through Maternal Marijuana Use: Predictions in Breastfed Infants	2023	Clinical Pharmacokinetics	Modeling	CBD	Infant exposure estimates	Very small exposures of CBD through milk predicted	[110]
3	Cannabidiol-Only Product Use in Pregnancy in the United States and Canada: Findings From the International Cannabis Policy Study	2024	Obstetrics & Gynecology	Survey	CBD	Real-world CBD use	Widespread use despite lack of safety data	[15]
4	Placental disposition of cannabidiol: An ex vivo perfusion study	2023	Epilepsia	Ex vivo	CBD	CBD placental distribution profile	Placenta acts as a depot compartment for CBD	[99]
5	Impact of prenatal exposure to Δ^9^-tetrahydrocannabinol and cannabidiol on birth size and postnatal growth trajectories	2025	Pediatric Obesity	Prospective cohort	CBD + THC	Exposure-birth size/growth association	Prenatal THC (but not CBD) exposure associated with fetal growth restriction	[98]

## Data Availability

No new data were created or analyzed in this study. Data sharing is not applicable to this article.
